# Journalistic Notes

**Published:** 1890-07

**Authors:** 


### Journalistic Notes.

THIS new weekly, *The Illustrated American*, is getting handsomely down to its work. Regarding the pictures of the scenes in Louisville after the great tornado, the beholder has the assurance that the paper reproduces the scenes exactly as caught by the photographer's plates. For sale by all newsdealers, twenty-five cents. Annual subscription, $10.00, less than twenty cents per copy.

---

THE *Medical News* is informed that upon the completion of the July issue Dr. I. Minis Hays will retire from the editorial chair of the *American Journal of the Medical Sciences*, and that Dr. Edward P. Davis will be his successor. With Dr. Hobart A. Hare in editorial charge of the *News*, and Dr. Davis installed as editor of the *American Journal of Medical Sciences*, the Lea's can boast of as strong a journalistic team as the country can produce.

---

THE *Annals of Gynecology and Pediatry* (formerly *Annals of Gynecology*) is now published by the *University of Pennsylvania Press*, but still under the editorship of Dr. E. W. Cushing, of Boston. We regret the change in the size of this journal, for anything but a standard octavo is difficult to bind in a convenient manner. It is, however, a beautiful specimen of magazine printing, and the *University Press* may well feel proud of its accomplished manager, Dr. A. L. Hummel.

One of the brightest women in New York, Mrs. Isabel Mallon, who, perhaps, knows more about woman's dress and fixings than any woman in America, has been added to the editorial staff of the *Ladies' Home Journal*, of Philadelphia. Mrs. Mallon is an experienced editorial writer, and will conduct one of the fullest and strongest fashion departments in the *Journal* ever attempted in a general magazine. Her new position makes her the best paid fashion writer in the country. Mrs. Mallon is young, pretty, and one of the best known women in New York society.

